# An Improvement of Shotgun Proteomics Analysis by Adding Next-Generation Sequencing Transcriptome Data in Orange

**DOI:** 10.1371/journal.pone.0039494

**Published:** 2012-06-29

**Authors:** Jiaping Song, Renjie Sun, Dazhi Li, Fengji Tan, Xin Li, Pingping Jiang, Xinjie Huang, Liang Lin, Ziniu Deng, Yong Zhang

**Affiliations:** 1 The Shenzhen Proteome Engineering Laboratory, BGI Shenzhen, Shenzhen, P. R. China; 2 National Center for Citrus Improvement, Hunan Agricultural University, Changsha, Hunan, P. R. China; George Mason University, United States of America

## Abstract

**Background:**

Shotgun proteomics data analysis usually relies on database search. Because commonly employed protein sequence databases of most species do not contain sufficient protein information, the application of shotgun proteomics to the research of protein sequence profile remains a big challenge, especially to the species whose genome has not been sequenced yet.

**Methodology/Principal Findings:**

In this paper, we present a workflow with integrated database to partly address this problem. First, we downloaded the homologous species database. Next, we identified the transcriptome of the sample, created a protein sequence database based on the transcriptome data, and integtrated it with homologous species database. Lastly, we developed a workflow for identifying peptides simultaneously from shotgun proteomics data.

**Conclusions/Significance:**

We used datasets from orange leaves samples to demonstrate our workflow. The results showed that the integrated database had great advantage on orange shotgun proteomics data analysis compared to the homologous species database, an 18.5% increase in number of proteins identification.

## Introduction

Over the past decade, mass spectrometry (MS)-based shotgun proteomics has emerged as a high-throughput, unbiased method for the identification of proteins in complex samples [1], [2]. Its application holds great potential in identifying comprehensive proteins profile in all kinds of species [3], [4]. Brechenmacher, L. analyzed the proteome of isolated soybean root hair cells using shotgun proteomics approaches. A complementary shotgun analysis identified 1,134 total proteins. The data presented provide useful insight into the metabolic activities of a single, differentiated plant cell type [5]. To better understand light regulation of C(4) plant maize development, Shen, Z. investigated dynamic proteomic differences between maize seedlings using a label-free quantitative proteomics approach [6].

However, because shotgun proteomics data analysis usually relies on database search and because commonly employed protein sequence databases of most species do not contain sufficient protein information, the application of shotgun proteomics to the research of protein sequence profile remains a big challenge. The most reliable method is to use homology species protein/gene-coding databases as a reference database for peptides search, which still has inherent defect in proteins identification. For example, to explore three main stages proteomics of citrus fruit development, Katz, E. established a comprehensive sequence database created by merging three major sources of sequences [7]. Lucker, J. developed a predicted grape peptide database for MS/MS applications derived from EST data using advanced clustering and trimming approaches and implemented for quantitative shotgun proteome profiling [8].

In this paper, we present a workflow with integrated database to partly address this problem. First, we downloaded the homologous species database. Next, we identified the transcriptome of the sample, created a protein sequence database based on the transcriptome data, and integtrated it with homologous species database. The last, we developed a workflow for identifying peptides simultaneously from shotgun proteomics data. We used datasets from orange leaves samples to demonstrate our workflow.

We finally increased the 18.5% proteins identified by using the integrated database, compared to traditional homologues database strategy.

## Methods

### Transcriptome Sequencing

Orange leaves were used in all experiments. Total RNA was isolated with TRIzol (Invitrogen) from each sample according to the manufacturer’s instructions. It was treated with RNase-free DNase I for 30 min at 37°C (New England BioLabs) to remove residual DNA.

**Figure 1:**
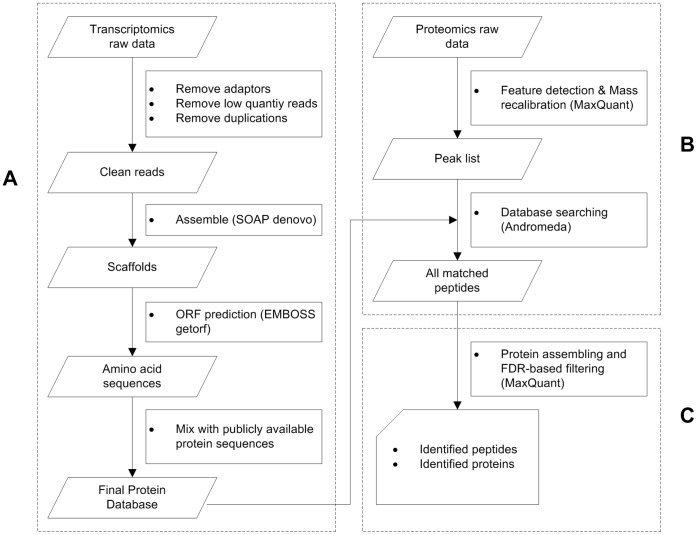
Workflow for identifying peptides based on integrated database.

Beads with oligo(dT) were used to isolate poly(A) mRNA. First-strand cDNA was synthesized using random hexamer-primer and reverse transcriptase (Invitrogen). The second-strand cDNA was synthesized using RNase H (Invitrogen) and DNA polymerase I (New England BioLabs). Then the cDNA libraries were prepared according to Illumina’s protocols, and were sequenced on the Illumina GA platform for 35 cycles.

The transcriptome sequence was assembled with short reads using SOAPdenovo software [9] (http://soap.genomics.org.cn), which adopts the de Bruijn graph data structure to construct contigs [10]. The reads were then realigned to the contig sequence, and the paired-end relationship between the reads was transferred to linkage between contigs. We constructed scaffolds starting with short paired-ends and then iterated the scaffolding process, step by step, using longer insert size paired-ends. To fill the intra-scaffold gaps, we used the paired-end information to retrieve read pairs that had one read well-aligned on the contigs and another read located in the gap region, then did a local assembly for the collected reads.

### Proteome Reference Database

First, we downloaded the homologous species database, clementine database (http://phytozome.net/clementine).

And then, based on scaffold data from transcriptome, reference database was processed using getorf of EMBOSS (version 6.3.1). Minimum nucleotide size of ORF to report is 500. [11].

**Figure 2:**
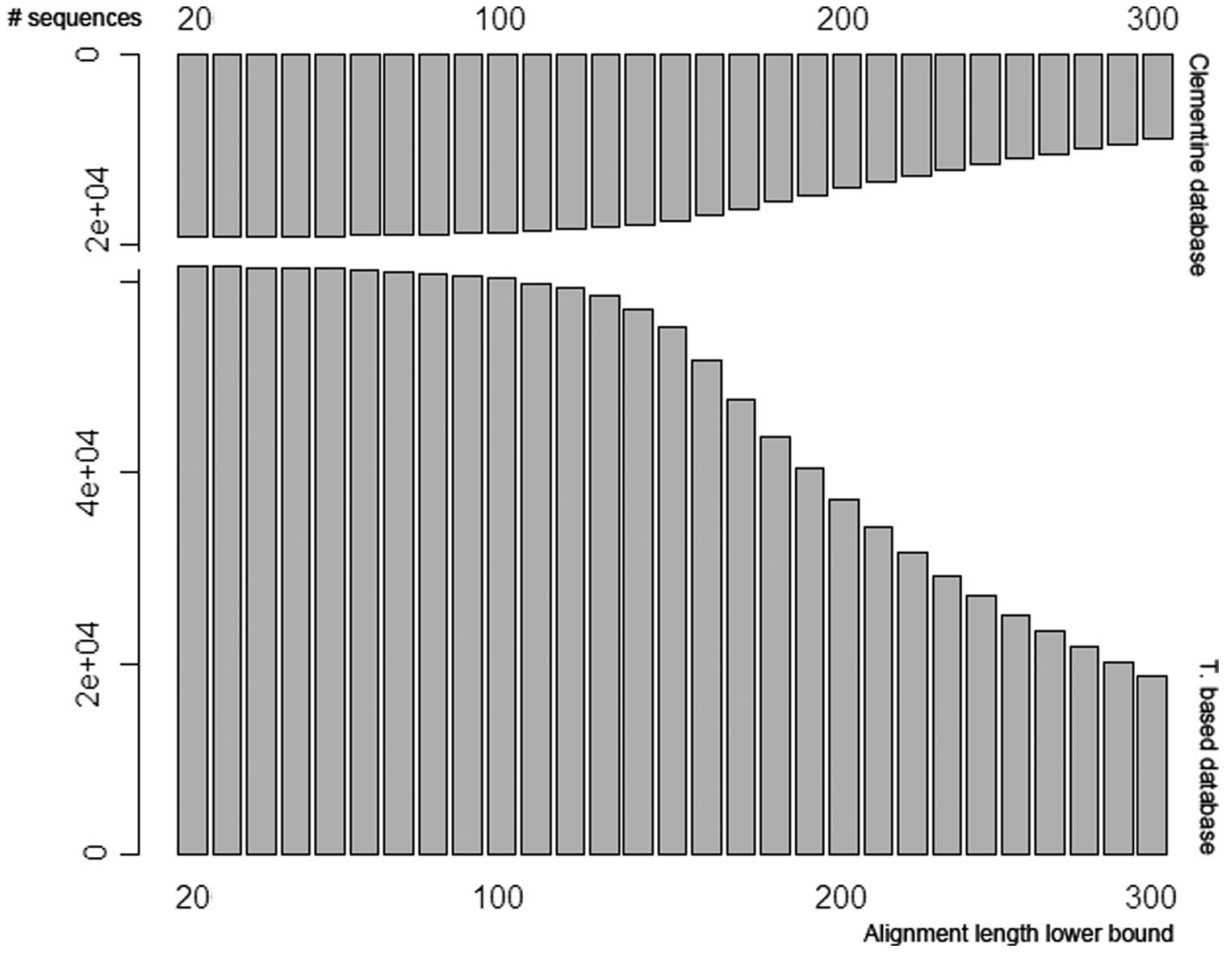
Number of aligned sequences between two databases based different alignment length threshold. The upper is the aligned sequences number of clementine database, the downer is the aligned sequences number of orange database.

Finally, transcriptome-based database were integrated to homologous species database, and proteome reference database for proteins identification was completed.

### Peptides Preparation and LC-MS

#### Protein Extraction

Leaves of orange were used in this study. Leaves were ground in liquid nitrogen. The powder was precipitated in a 10% (w/v) TCA, acetone solution containing 40 mM DTT at 20°C for 2 h. After centrifugation at 18,500 g for 1 h, the supernatant was discarded, and the pellet was rinsed with 20°C acetone containing 40 mM DTT. The final pellet was vacuum-dried and solubilized in 3 ml of 8 M (w/v) urea containing 2 M (w/v) thiourea, 40 mM DTT, 1 mM PMSF, 0.2 mM Na_2_VO_3_, and 1 mM NaF on ice for about 1 h. Insoluble material was removed by centrifugation at 18,500 g for 1 h. The protein concentration was determined using the 2-D Quant kit (GE Healthcare) with BSA as a standard. Samples were frozen in liquid nitrogen and stored at −80°C for further experiments.

**Figure 3:**
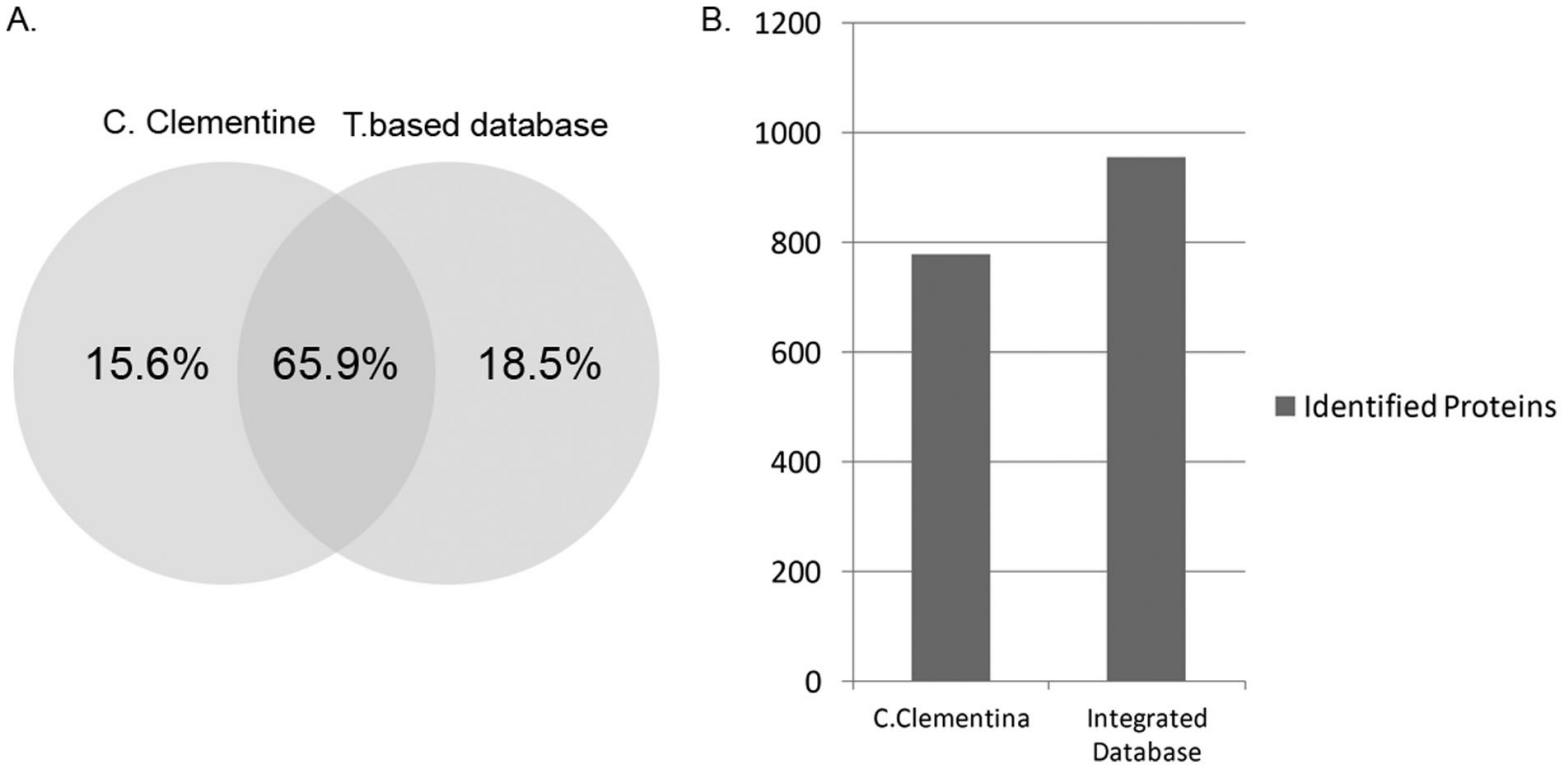
Identification comparison between homologous database and transcriptome based database (T. based database). (A) Venn chart for distribution of the proteins identified by MaxQuant based on two databases. (B) Numbers of proteins identification based on homologous and integrated database.

#### Protein Digestion

Protein digestion was performed as described previously [12]. After adjusting the pH to 8.5 with 1 M ammonium bicarbonate, total protein extracted from each sample was chemically reduced for 45 min at 55°C by adding DTT to 10 mM and carboxyamidomethylated in 55 mM iodoacetamide for 30 min at room temperature in the dark. Then trypsin (Roche Applied Science) was added to a final substrate/enzyme ratio of 30∶1 (w/w). The trypsin digest was incubated at 37°C for 12 h. After digestion, the peptide mixture was acidified by 10 µl of formic acid for further MS analysis. Samples not immediately analyzed were stored at −80°C.

**Figure 4:**
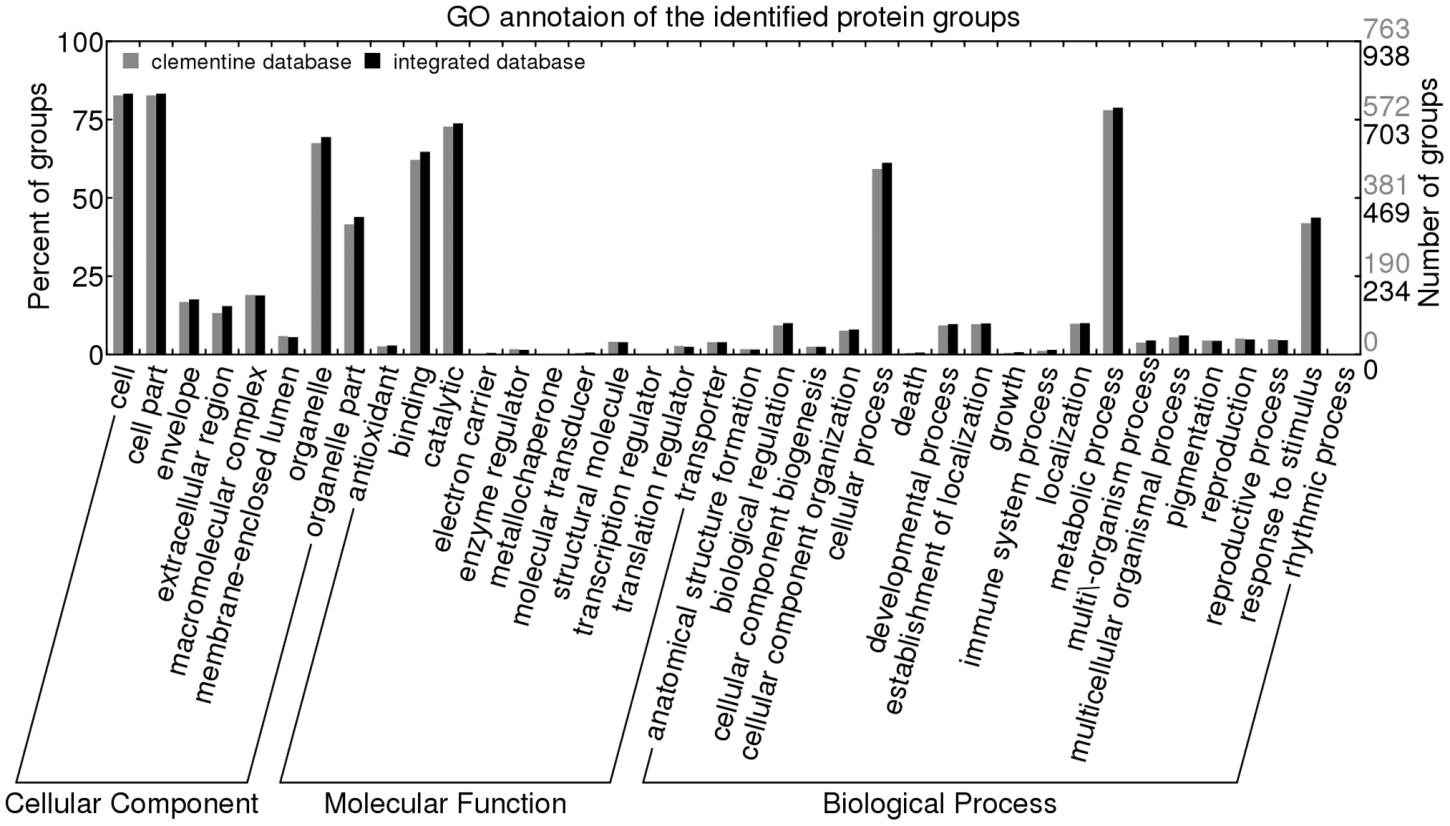
Distribution (by number and percentage) of protein groups identified with homologous database and integrated database into relevant secondary level GO classes, based on GO annotation.

#### LC-MS/MS analysis

The digestion mixtures were desalted by Strata X C18 column (Phenomenex) and vacuum-dried. Each samples were resuspended in certain volume of buffer A (2% ACN, 0.1%FA) and centrifuged at 20000 g for 10 min. In each sample, the final concentration of peptide was about 0.5 ug/ul on average. 10 ul supernatant was loaded on an Shimadzu LC-20AD nanoHPLC by the autosampler onto a 2 cm C18 trap column (inner diameter 200 µm) and the peptides were eluted onto a resolving 10 cm analytical C18 column (inner diameter 75 µm) made in-house. The samples were loaded at 15 µL/min for 4 min, then the 91 min gradient was run at 400 nL/min starting from 2 to 35% B (98%ACN, 0.1%FA), followed by 5 min linear gradient to 80%, and maintenance at 80% B for 8 min, and finally return to 2% in 2 min.

The peptides were subjected to nanoelectrospray ionization followed by tandem mass spectrometry (MS/MS) in an LTQ Orbitrap Velos (Thermo) coupled online to the HPLC. Intact peptides were detected in the Orbitrap at a resolution of 60 000. Peptides were selected for MS/MS using collision induced dissociation (CID) operating mode with a normalized collision energy setting of 35%; ion fragments were detected in the LTQ. A data-dependent procedure that alternated between one MS scan followed by ten MS/MS scans was applied for the ten most abundant precursor ions above a threshold ion count of 5000 in the MS survey scan.

### Data Processing and Protein Identification

Continuum LC-MS data were processed and searched using MaxQuant software (version 1.1.1.36) [13], [14]. Raw data sets were processed including ion detection, de-isotoping, de-convolution, and peak lists generated based on the assignment of precursor ions and fragments based on similar retention times. The principles of the applied data clustering and normalization have been explained previously in great detail [15], [16].

A false discovery rate (FDR) of 0.01 for proteins and peptides and a minimum peptide length of 6 amino acids were required. The mass accuracy of the precursor ions was improved by the time-dependent recalibration algorithm of MaxQuant. The Andromeda search engine was used to search the MS/MS spectra against database combined with 262 common contaminants and concatenated with the reversed versions of all sequences. Enzyme specicicity was set to trypsin specificity, allowing cleavage N-terminal to proline. Further modifications were cysteine carbamidomethylation (fixed) as well as protein N-terminal acetylation and methionine oxidation (variable). MaxQuant was used for scoring of the peptides for identification. A maximum of two missed cleavages were allowed. Peptide identification was based on a search with an initialmass deviation of the precursor ion of up to 7 ppm. The fragment mass tolerance was set to 20 ppm on the m/z scale.

### Gene Ontology Annotation

In order to characterize the identified proteins in terms of biological functions, we aligned them against plant portion of NCBI nr database released at 20110525 utilizing NCBI blastp algorithm [17], with evalue set to 1e-5. Gene Ontology annotations are assigned to the sequences using blast2go algorithm [18] version 2.3.5.

## Results and Discussion

### Setup of the Workflow

As illustrated in Fig. 1, to the species whose peptides database do not contain sufficient information, our workflow for identifying peptides based on shotgun proteomics data includes three steps: transcriptome identification, database creation and peptide identification.

The protein sequence database was created based on the transcriptome data and the homologous species database (Fig. 1A). To obtain a global view of the orange transcriptome, we performed high-throughput RNA-seq, using Illumina sequencing technology, on poly(A)-enriched RNAs from orange leaves. To minimize the likelihood of systematic biases in transcriptome sampling, multiple cDNA libraries were prepared and data were generated from three paired-end libraries with insert sizes ranging from 100 to 500 base pairs (bp). We conducted in-depth sequencing by paired-end RNAseq on the three samples.

The reads were then realigned to the contig sequence, and the paired-end relationship between the reads was transferred to linkage between contigs. We constructed scaffolds starting with short paired-ends and then iterated the scaffolding process, step by step, using longer insert size paired-ends. To fill the intra-scaffold gaps, we used the paired-end information to retrieve read pairs that had one read well-aligned on the contigs and another read located in the gap region, then did a local assembly for the collected reads.

Based on scaffold data from transcriptome, reference database was processed using getorf of EMBOSS (version 6.3.1). Minimum nucleotide size of ORF to report is 500. The created database contains 70,134 entries. Transcriptome-based database were integrated to homologous species database, a downloaded clementine database (http://phytozome.net/clementine; 32,473 entries), and the proteome reference database for proteins identification was completed. The analysis between two databases would be discussed below.

After integration of the database, shotgun proteomics data can be searched against the database using a database search engine (Fig.1B). The next important step is the confidence evaluation of the peptide identifications, i.e. FDR estimation. A FDR of 0.01 for proteins and peptides and a minimum peptide length of 6 amino acids were required.

In the last step of the workflow, the peptides were identified based on the refined separate FDR estimation and an easily interpretable report was generated. (Fig.1C).

### Application on Orange Leaves Data Sets

With the procedure described above, we performed database search and peptide identification for data sets from orange leaves. An orange homologous database (clementine database; http://phytozome.net/clementine; 32,473 entries) integrated with transcriptome-based database (70,134 entries). The integrated database was used for peptides identification.

Here we noticed that there were twice as many entries from orange leaf transcriptome-based database as from clementine database. To gain better understanding of the similarity of the sequences from the two databases, we aligned clementine database against the orange transcriptome-based database, utilizing the NCBI blastp algorithm [17] with e-value threshold set to 1e-5. Blastp output was subjected to filtering by requiring that two sequences had alignment >20 amino acids with >90% identity.

The result was that 19, 177 out of 32, 473 (59.06%) clementine sequence and 57, 268 out of 70, 134 (81.66%) orange transcriptome-based sequences can be considered sufficiently similar. The ratio of the two numbers, approximately 0.33∶1, implicated that three orange sequences corresponded to one clementine sequence roughly.

By increasing the alignment length threshold from 20 amino acids upwards to 300 in steps of 10, we had generally decreasing number of sequences involved in alignment (Fig.2). The different decreasing rates of the aligned sequences number reflected the corresponding distribution of alignment length.

The results showed that high throughput sequencing transcriptome data were more comprehensive, the integrated database could increase the numbers of identified peptides.

MaxQuant was used as the search engine, and the FDR threshold was set to 0.01. Thus, 2951 unique peptides were identified, which were mapped to 955 indiscernible protein groups. The number of protein groups was 778 and 806 separately, based on different reference database (Fig.3A), corresponding to 81.47%, and 84.40% of all protein groups identified.

The results showed that the integrated database had great advantage on orange shotgun proteomics data analysis compared to the Homologous species database, 18.5% increase in number of proteins identification (Fig.3B).

In order to know whether identified protein groups differ in terms of GO categorization, we compared these two using WEGO [19] algorithm (Fig.4). All of the identified proteins were classified into 38 different functional categories and subcategories. The results showed that no significant difference between them, which illustrated that the increased indentified proteins were similar to the original in functional categorization.

In summary, we have presented a workflow with integrated database for the peptides identification, which will be useful to the proteome research of species whose protein sequence database is defective. Recently, more and more big next-generation sequencing projects were launched, such as 1,000 Plant and Animal Genome Project, 1,000 Plant Transcriptome Project. The workflow will help the scientists who are working on any species even without original protein database. The number of proteins identified could be 2 times of the past studies for some species. We believe that more proteome studies will be performed well by using our strategy.
