# Correction: An Improvement of Shotgun Proteomics Analysis by Adding Next-Generation Sequencing Transcriptome Data in Orange

**DOI:** 10.1371/annotation/920bd689-3af7-418f-8149-43e683e18852

**Published:** 2012-10-08

**Authors:** Jiaping Song, Renjie Sun, Dazhi Li, Fengji Tan, Xin Li, Pingping Jiang, Xinjie Huang, Liang Lin, Ziniu Deng, Yong Zhang

The affiliation for the tenth author was incorrect. Yong Zhang is not affiliated with #2 but with #1 The Shenzhen Proteome Engineering Laboratory, BGI Shenzhen, Shenzhen, P. R. China. The PDF version of the article is correct. 

